# l‐tetrahydropalmatine suppresses osteoclastogenesis in vivo and in vitro via blocking RANK‐TRAF6 interactions and inhibiting NF‐κB and MAPK pathways

**DOI:** 10.1111/jcmm.14790

**Published:** 2019-11-14

**Authors:** Xin Zhi, Lipeng Wang, Huiwen Chen, Chao Fang, Jin Cui, Yan Hu, Liehu Cao, Weizong Weng, Qirong Zhou, Longjuan Qin, Hongyuan Song, Yajun Wang, Yao Wang, Hao Jiang, Xiaoqun Li, Sicheng Wang, Xiao Chen, Jiacan Su

**Affiliations:** ^1^ Department of Orthopedics Trauma Shanghai Changhai Hospital Naval Military Medical University Shanghai China; ^2^ Basic Medical School Naval Military Medical University Shanghai China; ^3^ Graduate Management Unit Shanghai Changhai Hospital Naval Military Medical University Shanghai China; ^4^ Orthopedic Basic and Translational Research Center Jiangyin China; ^5^ Department of Ophthalmology Shanghai Changhai Hospital Naval Military Medical University Shanghai China; ^6^ Department of Orthopedics Shanghai Zhongye Hospital Shanghai China; ^7^ Department of Chemistry Fudan University Shanghai China; ^8^ China‐South Korea Bioengineering Center Shanghai China

**Keywords:** l‐tetrahydropalmatine, osteoclastogenesis, osteoporosis, RANKL

## Abstract

Bone homeostasis is delicately orchestrated by osteoblasts and osteoclasts. Various pathological bone loss situations result from the overactivated osteoclastogenesis. Receptor activator of nuclear factor κB ligand (RANKL)‐activated NF‐κB and MAPK pathways is vital for osteoclastogenesis. Here, we for the first time explored the effects of l‐tetrahydropalmatine (l‐THP), an active alkaloid derived from corydalis, on the formation and function of osteoclasts in vitro and in vivo. In RAW264.7 cells and bone marrow monocytes cells (BMMCs), l‐THP inhibited osteoclastic differentiation at the early stage, down‐regulated transcription level of osteoclastogenesis‐related genes and impaired osteoclasts functions. Mechanically, Western blot showed that l‐THP inhibited the phosphorylation of P50, P65, IκB, ERK, JNK and P38, and the electrophoretic mobility shift assay (EMSA) revealed that DNA binding activity of NF‐κB was suppressed, ultimately inhibiting the expression of nuclear factor of activated T cells (NFATc1). Besides, Co‐immunoprecipitation indicated that l‐THP blocked the interactions of RANK and TNF receptor associated factor 6 (TRAF6) at an upstream site. In vivo, l‐THP significantly inhibited ovariectomy‐induced bone loss and osteoclastogenesis in mice. Collectively, our study demonstrated that l‐THP suppressed osteoclastogenesis by blocking RANK‐TRAF6 interactions and inhibiting NF‐κB and MAPK pathways. l‐THP is a promising agent for treating osteoclastogenesis‐related diseases such as post‐menopausal osteoporosis.

## 1 INTRODUCTION

The balance of bone metabolism is delicately regulated by bone formation and bone resorption.^1^ Destruction of bone homeostasis which is maintained by osteoblasts and osteoclasts causes pathological bone loss in many bone diseases, such as post‐menopausal osteoporosis (PMOP) and rheumatoid arthritis (RA).^2^ To inhibit osteoclastogenesis and osteoclast function remains an important treatment strategy of bone loss diseases.^3^ Previous studies have shown that osteoprotegerin binds to RANKL, blocking the effects of RANKL in vitro and in vivo, ultimately inhibiting the differentiation of osteoclasts.^4^, ^5^, ^6^

PMOP is a classic orthopaedic disorder resulting from overactivated osteoclasts.^7^ The previous study demonstrated that oestrogen could exert anti‐osteoporosis effect by stimulating osteoblasts to secrete osteoprotegerin (OPG) and inhibiting osteoclastogenesis.^8^, ^9^ The oestrogen withdrawal after menopause leads to the overactivation of osteoclasts, which further leads to the increased bone turnover rate and net bone loss.^7^, ^10^

Osteoclastogenesis is regulated by several cytokines and signalling pathways. RANK (receptor activator of NF‐κB) and its ligand RANKL are essential for osteoclastogenesis. RANKL binds to RANK on osteoclast precursors to recruit TNF receptor‐associated factors 2, 3, 5, 6 (TRAF 2, 3, 5, 6) to initiate osteoclast differentiation.^11^ And TRAF6 would further stimulate the phosphorylation of P50, P65, IκB (NF‐κB pathway), ERK, JNK and P38 (MAPK pathway). The master transcription factor for osteoclastogenesis, nuclear factor of activated T cells (NFATc1), is activated.^12^ Osteoclastogenesis‐related genes like Cathepsin K, tartrate‐resistant acid phosphatase (TRAP), calcitonin receptor (CTR) and matrix metallopeptidase 9 (MMP9) are increased. It has been verified that selective inhibition of NF‐κB and MAPK pathways could inhibit osteoclastogenesis.^13^

l‐tetrahydropalmatine (l‐THP), an active component derived from the corydalis, is a natural component with anti‐inflammation effects.^14^ Some recent studies have indicated that l‐THP could suppress chronic inflammation in animal models.^15^, ^16^ And l‐THP could protect the rat against D–galactose induced memory impairment through the inhibition of the NF‐κB pathway.^17^ We hypothesized that l‐THP could inhibit osteoclastogenesis. In order to verify this speculation, we evaluated the effects of l‐THP on RANKL‐induced osteoclastogenesis in vitro demonstrated by bone marrow monocytes and RAW 264.7 cells, and ovariectomy (OVX)‐induced bone loss in vivo and further explored the underlying mechanism. We showed that l‐THP ameliorated bone loss in ovariectomized mice and effectively inhibited RANKL‐induced osteoclastogenesis in vitro with little influence on osteogenesis and adipogenesis. For the underlying mechanism, l‐THP inhibited NF‐κB and MAPK pathways through interrupting the interaction between RANK and TRAF6.

## 2 METHODS

### 2.1 Reagents

**Figure 1:**
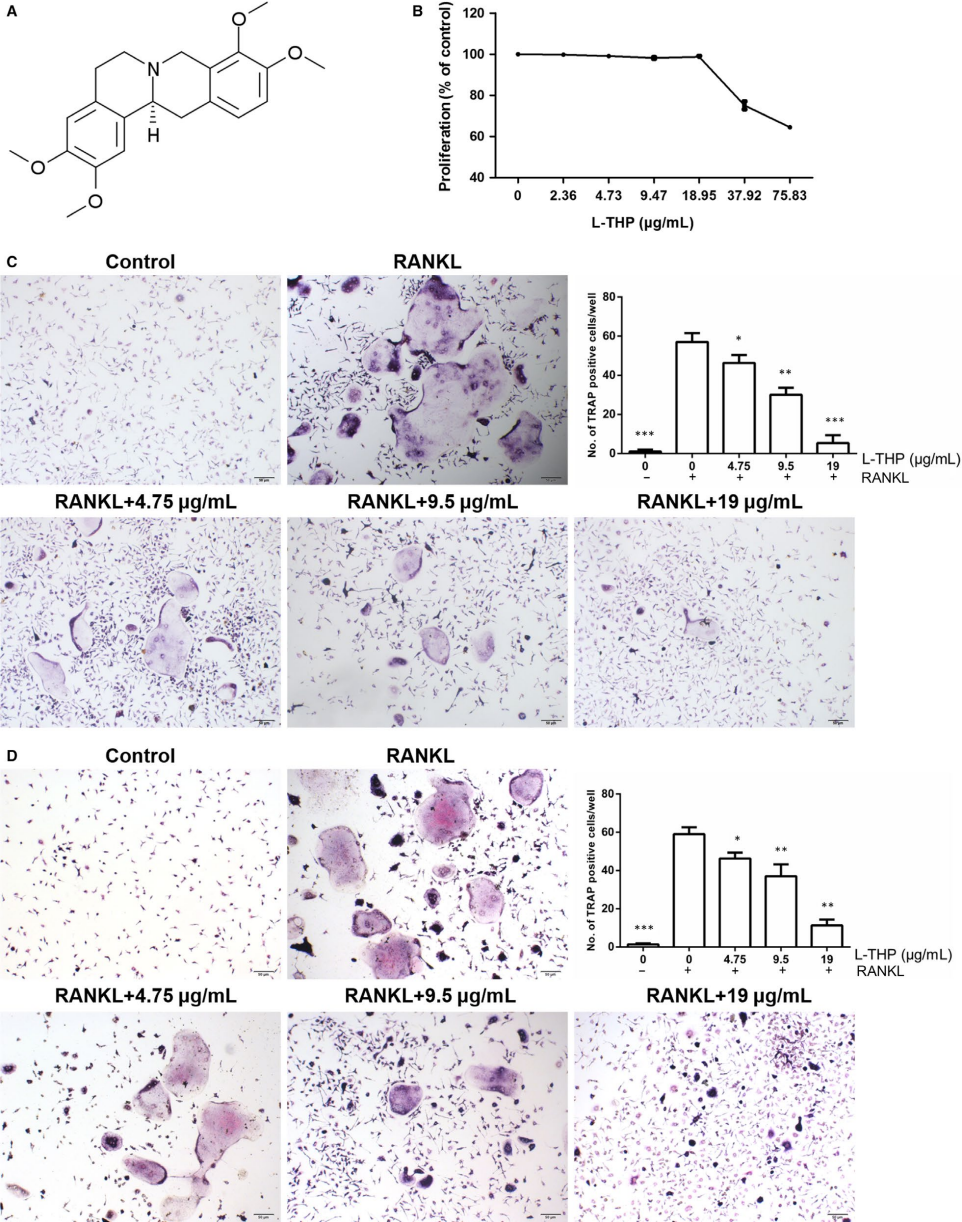
l‐tetrahydropalmatine (l‐THP) inhibits osteoclastogenesis in vitro. A, Chemical structure of l‐THP. B, BMMCs were seeded and cultured for 24 h, then treated with l‐THP (0, 2.36, 4.73, 9.47, 18.95, 37.92 and 75.83 μg/mL). After 72 hours, the MTT solution was added and the absorbance at 490 nm was detected. C, BMMCs stimulated by 30 ng/mL M‐CSF, 50 ng/mL RANKL were fixed and stained by TRAP staining kits to detect the formation of TRAP‐positive cells. D, RAW264.7 cells stimulated by 30 ng/mL M‐CSF, 50 ng/mL RANKL were fixed and stained by TRAP staining kits to detect the formation of TRAP‐positive cells. (*P < .05, **P < .01, ***P < .001)

l‐tetrahydropalmatine (Figure 1A) was purchased from Standard Technical Service Company (Shanghai, China). Dimethyl sulfoxide (DMSO) was bought from Solarbio (Beijing, China). l‐THP was dissolved in the solution comprised of 2% DMSO and 0.9% normal saline for further use. Foetal bovine serum and trypsin were purchased from HyClone (Logan, UT, USA). High‐glucose DMEM medium, α‐MEM, penicillin and streptomycin were purchased from R&D (MN, USA).

### 2.2 Animal model and experimental procedures

Animal modelling and experimental procedures were conducted in the laboratory of Shanghai Changhai Hospital (SPF Grade, SYXK 2015‐0017). C57BL/6 mice were offered by Weitonglihua Co. All animal procedures abided by the rules of the Animal Research Ethical Committee of the Changhai hospital.

C57BL/6 mice (8 weeks old, female) were distributed into 3 equal groups (n = 6/group) randomly: Sham group and OVX group administered with DMSO vehicle, OVX group administered with l‐THP (4 mg/kg/d, intraperitoneally injected) dissolved in DMSO. The mice were anaesthetized with 5% chloral hydrate (Sigma‐Aldrich). Both sides of ovaries and parts of the oviducts were resected, while the mice from the sham group received an operation to surgically isolate and expose bilateral ovaries without resection. All mice were permitted to recover for 24 hours after the operation. After 6 weeks of intervention, the femurs and blood were collected from anaesthetized mice. Then the mice were killed by an overdose of 5% chloral hydrate.

### 2.3 Bone histomorphometric analysis

Bone histomorphometric analysis was applied to measure the trabecular bone area and osteoclasts number in the femoral metaphysis. The femurs were fixed by 40% formaldehyde (Sigma‐Aldrich) followed by 2‐week decalcification by 10% EDTA solution (Solarbio). The femurs were sliced into 4‐μm‐thick sections with a microtome. Prepared sections were then stained with haematoxylin and eosin (H&E staining), followed by TRAP staining (Sigma‐Aldrich).

### 2.4 Micro CT scanning and analysis

We used high solution X‐ray microcomputed tomography (Skyscan) with the conditions (8 μm of resolution, 80 kV of the voltage and 124 μA of the electric current) to evaluate the femoral trabecular from 100 layers beneath the epiphyseal plate. The following parameters were used, including the bone mineral density (BMD), bone volume/total volume (BV/TV), trabecular number (Tb.N) and bone surface area/total volume (BS/TV).

### 2.5 Serum biochemistry

After being anaesthetized, the left eyeball of the mouse was resected to collect the blood via fundus venous bleeding. To isolate and obtain the serum, the blood was kept still for 30 minutes and then centrifuged at 1000 *g* for 30 minutes. ELISA kits (R&D Systems) were used to evaluate the levels of C‐terminal telopeptide‐1 (CTX‐1), tumour necrosis factor α (TNF‐α), Interleukin 6 (IL‐6) and tartrate‐resistant acid phosphatase 5b (TRACP 5b) in the serum.

### 2.6 MTT assay

We conducted an MTT (R&D Systems) assay to detect the l‐THP cytotoxic effect on BMMCs according to the manufacturer's protocols. Cells were cultured and seeded onto a 96‐well plate. After 24 hours, cells were treated with l‐THP (0, 2.36, 4.73, 9.47, 18.95, 37.92 and 75.83 μg/mL). After 72 hours of incubation, the MTT solution was added to all wells. The absorbance at 490 nm was detected by a microplate reader.

### 2.7 In vitro osteogenesis and adipogenesis assay

To identify the role of l‐THP on osteogenesis and the formation of the calcified nodule, we flushed bilateral femoral bone marrow of 4‐week‐old C57BL/6 mice to isolate bone marrow mesenchymal stem cells (BMSCs). To induce osteogenesis, BMSCs were cultured with complete medium supplied with 100 nmol/L dexamethasone, 50 μmol/L ascorbic acid and 10 mmol/L β‐glycerophosphate (Cyagen Biosciences). Prepared cells were stained with ALP staining (Sigma‐Aldrich) after osteogenic induction for 14 days, while alizarin red staining was conducted after 21 days. To induce adipogenesis, BMSCs were cultured with 10% FBS α‐MEM supplied with 10 μg/mL insulin, 200 μmol/L indomethacin, 1 μmol/L dexamethasone and 0.5 mmol/L 3‐isobutyl‐1‐methylxanthine (IBMX) (Cyagen Biosciences). Differentiated cells were then marked with Oil Red O staining (Sigma‐Aldrich).

### 2.8 In vitro osteoclastogenesis assay

RAW264.7 cells were purchased from the Shanghai Academy of Chinese Sciences. Bone marrow monocytes (BMMCs) were harvested from bilateral femur marrow following the same method as BMSCs were harvested. Then cells were stimulated into osteoclastogenesis induced by 30 ng/mL macrophage colony‐stimulating factor (M‐CSF, R&D) and 50 ng/mL RANKL (R&D), with or without l‐THP (0, 4.75, 9.50, 19.00 μg/mL). RAW264.7 cells were also stimulated into osteoclastogenesis by the same concentrations of M‐CSF and RANKL and incubated with the same concentrations of l‐THP. After 7 days, all the cells were stained by a TRAP staining kit (Sigma‐Aldrich). Osteoclast cells were identified as large size cells with more than 3 nuclei. For F‐actin staining, RANKL‐induced RAW 264.7 cells were fixed with 4% formaldehyde solution for 15 minutes. Fixed cells were incubated with 0.5% TritonX‐100 for 10 minutes and then stained by phalloidin conjugated with rhodamine (Biotium).

### 2.9 Pit‐formation assay

RAW264.7 cells were cultured and induced by M‐CSF (30 ng/mL) and RANKL (50 ng/mL). After 7 days, osteoclasts were isolated by collagenase and seeded on a synthetic bio‐mimetic bone surface (Corning) with incubation of 50 ng/mL RANKL and 30 ng/mL M‐CSF, followed by treatment of l‐THP (0, 4.75, 9.50, 19.00 μg/mL). After treatment for 2 days, the plates were cleaned and air‐dried for 4 hours. The resorbed area was visualized using an optical microscope. The enumeration of pits was quantified using Image‐Pro Plus software.

### 2.10 Co‐immunoprecipitation

RAW264.7 cells were harvested after treatment with l‐THP (19.00 μg/mL) for 60 minutes after the induction of RANKL (50 ng/mL). Cells were subjected to homogenization with IP buffer and a micro pestle. After gentle shaking, cell lysate was centrifuged at 4°C for 30 minutes at 14000 *g*. The sediment was discarded, and the supernatant was collected. The supernatant was separated and mixed with beads bounded to the specific antibody (TRAF6, Santa Cruz) followed by incubation on a gentle‐shaking platform at 4°C for 24 hours. The mixture was then centrifuged for 10 minutes at 800 *g* at 4°C with the supernatant discarded. The remaining beads were washed thoroughly with IP washing buffer to collect the protein complex. Finally, the protein complex was boiled for further sulphate‐polyacrylamide gel electrophoresis (SDS‐PAGE) and Western blotting analysis.

### 2.11 Immunofluorescence staining

Immunofluorescence staining was applied to determine the effects on the P65 translocation in RAW264.7 cells. In short, cells were fixed with 40% formaldehyde, then washed by Triton X‐100, followed by incubation with anti‐P65 or anti‐F‐actin antibody, goat antimouse IgG antibody and fluorescein‐conjugated streptavidin (Santa Cruz).

### 2.12 Electrophoretic mobility shift assay

An EMSA was conducted for measuring the quantity of DNA binding of NF‐κB. BMMCs were prepared by incubation with RANKL (0 or 30 minutes) or RANKL plus l‐THP (19.00 μg/mL, 0 or 30 minutes). Cells were then washed by PBS three times and the adherent cells were collected by scraping off the culture dish and were centrifuged. The cells were then resuspended with a 32P‐labelled DNA duplex (Beyotime Biotechnology). The mixture was then run in sodium dodecyl SDS‐PAGE system at 4°C for 4 hours.

### 2.13 Western blotting

Western blotting was used to determine the activation level of MAPK and NF‐κB pathway by the treatment of l‐THP. In short, RAW264.7 cells were treated by l‐THP (19.00 μg/mL) together with RANKL for 0, 15, 30 and 60 minutes. The levels of phosphorylated ERK, JNK, P38, P65, P50 and IκB were evaluated by Western blotting analysis. Briefly, RIPA Lysis and Extraction Buffer (Pierce Biotechnology, Rockford) was used to obtain the total protein from cell lysis. Protein samples were then quantified by BCA assay kit (Pierce Biotechnology, Rockford) and added into 12% SDS‐PAGE gels at equivalent amounts of 10 μg per lane. After electrophoresis, the protein was transferred to NC membranes. Then the NC membranes were incubated with 5% skim milk for 2 hours and washed by Tris‐buffered saline with Tween (TBST). Prepared membranes were incubated with specific primary antibodies overnight. Membranes were then washed by TBST 3 times followed by incubation with HRP‐conjugated secondary antibodies for 2 hours. Finally, chemiluminescence was used to detect the protein bands which were imaged by X‐ray films. Image J software was used to quantify the protein band intensities. Primary antibodies were monoclonal antibodies (Santa Cruz). Second antibodies were HRP‐conjugated rabbit antimouse antibody (Santa Cruz).

### 2.14 Quantitative real‐time polymerase chain reaction

Quantitative real‐time polymerase chain reaction (qRT‐PCR) was used to detect the transcription of osteoclastogenesis‐related genes. Briefly, cells were lysed using Trizol buffer to extract total RNA. Then the complementary DNA was generated from RNA using reverse transcriptase kit. SYBR Green dye and specific primers together detected the expression of the relevant gene. β‐Actin was detected as an internal reference. The mouse‐specific primers were described as below: mouse RANK (forward: 5′‐CTGCTCCTCTTCATCTCTGTG‐3′; reverse: 5′‐CTTCTGGAACCATCTTCTCCTC‐3′), C‐Fms (forward: 5′‐TTCACTCCGGTGGTGGTGGCCTGT‐3′; reverse: 5′‐GTTGAGTAGGTCTCCATAGCAGCA‐3′), NFATc1 (forward: 5′‐TGGAGAAGCAGAGCACAGAC‐3′; reverse: 5′‐GCGGAAAGGTGGTATCTCAA‐3′), Cathepsin K (forward: 5′‐GGGAGAAAAACCTGAAGC‐3′; reverse: 5′‐ATTCTGGGGACTCAGAGC‐3′), MMP9 (forward: 5′‐CGTGTCTGGAGATTCGACTTGA‐3′; reverse: 5′‐TTGGAAACTCACACGCCAGA‐3′), CTR (forward: 5′‐ GTCGATTGCTGCTTTGTTGCTTCC‐3′; reverse: 5′‐ GTGATGGCGTGGATAATGGTTGGC‐3′), TRAP (forward: 5′‐GCTGTCCTGGCTCAAAAAGC‐3′; reverse: 5′‐CACACCGTTCTCGTCCTGAA‐3′) and β‐actin (forward: 5′‐GTACGCCAACACAGTGCTG‐3′; reverse: 5′‐CGTCATACTCCTGCTTGCTG‐3′).

### 2.15 Overexpression of NFATc1

Lentivirus vector construction and targeted cells infection were conducted as described previously.^1^ Briefly, the cDNA products were prepared by RT‐PCR, and RNA was extracted from mouse brain tissue, using NFATc1 primers. The amplified products were then cloned into a pcDH1‐CMV lentiviral vector (System Biosciences). A pcDH1‐NFATcl expression vector was thus produced. The 293T virus packaging cell line was used to generate Lv‐NFATc1 after transfection with the expression vector. RAW264.7 cells were counted at 2 × 10^5^ cells/well and were cultured in 6‐well plates followed by adding Lv‐NFATc1. Osteoclastogenesis level was evaluated through TRAP staining after RANKL stimulation and treatment of l‐THP.

### 2.16 Statistical analysis

Data more than 3 groups were analysed using one‐way analysis of variance (ANOVA) analysis of the variance, while Student′s *t* test was used to compare two independent groups. All data were presented as the mean value ± SD. Statistical analysis was processed through SPSS software, version 21.0 (IBM). Statistically, significance was regarded when a *P*‐value < .05.

## 3 RESULTS

### 3.1 l‐THP inhibited osteoclastogenesis in vitro

Cytotoxic effect was examined by MTT assay. The result revealed that l‐THP had no cytotoxic effect with a concentration below 19.00 μg/mL (Figure 1B). To ensure the effect of l‐THP on osteoclastogenesis of BMMCs and RAW264.7 cells, we performed TRAP staining assay following the treatment of l‐THP (4.75, 9.50, 19.00 μg/mL). 7 days after the induction, the TRAP‐positive large size cells markedly increased in vehicle‐treated group, and l‐THP dose‐dependently suppressed osteoclastogenesis (Figure 1C&D).

### 3.2 l‐THP suppressed osteoclast function and osteoclastogenesis relevant genes in vitro

**Figure 2:**
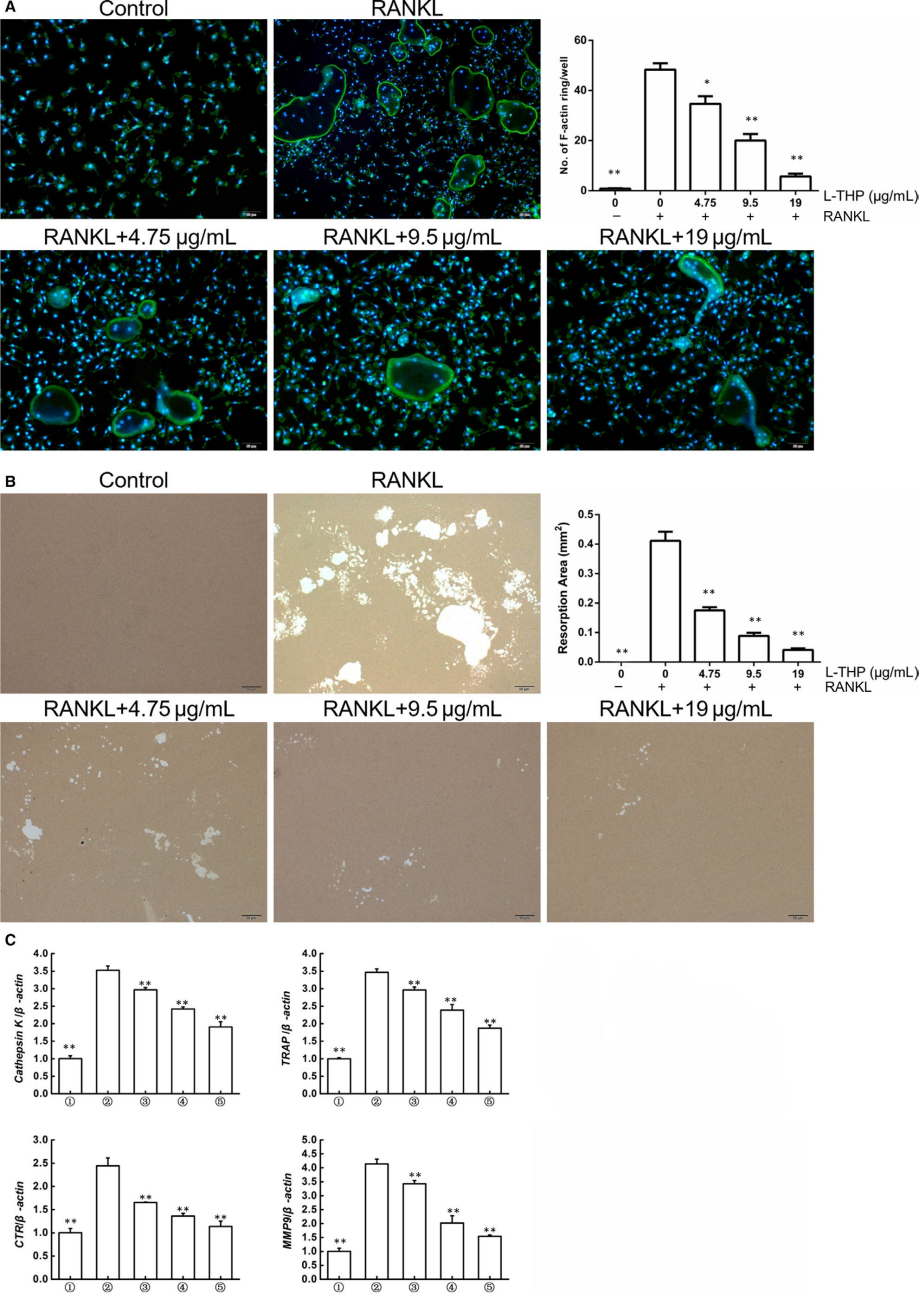
l‐tetrahydropalmatine (l‐THP) inhibits osteoclast function in vitro. A, Induced RAW264.7 cells were stained by phalloidin conjugated with rhodamine to detect F‐actin. B, RAW264.7 cells induced by M‐CSF (30 ng/mL) and RANKL (50 ng/mL) for 7 d, and then cells were isolated by collagenase and seeded on a synthetic bio‐mimetic bone surface with incubation of 50 ng/mL RANKL and 30 ng/mL M‐CSF, followed by treatment of l‐THP for 2 days (0, 4.75, 9.50 and 19.00 μg/mL). Then the resorbed area was visualized and quantified. C, RAW 264.7 cells were stimulated by M‐CSF (30 ng/mL) and RANKL (50 ng/mL) for 7 days, and the total RNA was extracted for RT‐PCR of Cathepsin K, TRAP, CTR and MMP9 with β‐actin as the internal control. Groups in the figures are divided as follows: 1. RAW264.7 cells; 2. RAW264.7 cells induced with M‐CSF (30 ng/mL), RANKL (50 ng/mL) and PBS; 3. RAW264.7 cells induced with M‐CSF (30 ng/mL), RANKL (50 ng/mL) and treated with 4.75 μg/mL l‐THP; 4. RAW264.7 cells induced with M‐CSF (30 ng/mL), RANKL (50 ng/mL) and treated with 9.5 μg/mL l‐THP; 5. RAW264.7 cells induced with M‐CSF (30 ng/mL), RANKL (50 ng/mL) and treated with 19 μg/mL l‐THP (*P < .05, **P < .01 vs Group 2)

We tried to detect whether l‐THP altered actin ring formation, which is essential for osteoclast functions. The results revealed that upon induced by RANKL, RAW264.7 cells exhibited obvious actin ring formation and the shape of mature osteoclasts. However, l‐THP (4.75, 9.50, 19.00 μg/mL) potently inhibited this process demonstrated by the decreased number and smaller size of the actin ring (Figure 2A). We next examined whether osteolytic pit formation was altered by the treatment of l‐THP (Figure 2B). We observed that the resorbed area on the bio‐mimetic synthetic surface reduced significantly by the treatment of l‐THP.

Furthermore, RT‐PCR demonstrated that the osteoclastogenesis relevant genes (cathepsin K, TRAP, CTR and MMP9) were up‐regulated after RANKL stimulation, while their mRNA levels significantly decreased by the treatment of l‐THP (Figure 2C).

### 3.3 l‐THP inhibited osteoclast differentiation at the early stage

**Figure 3:**
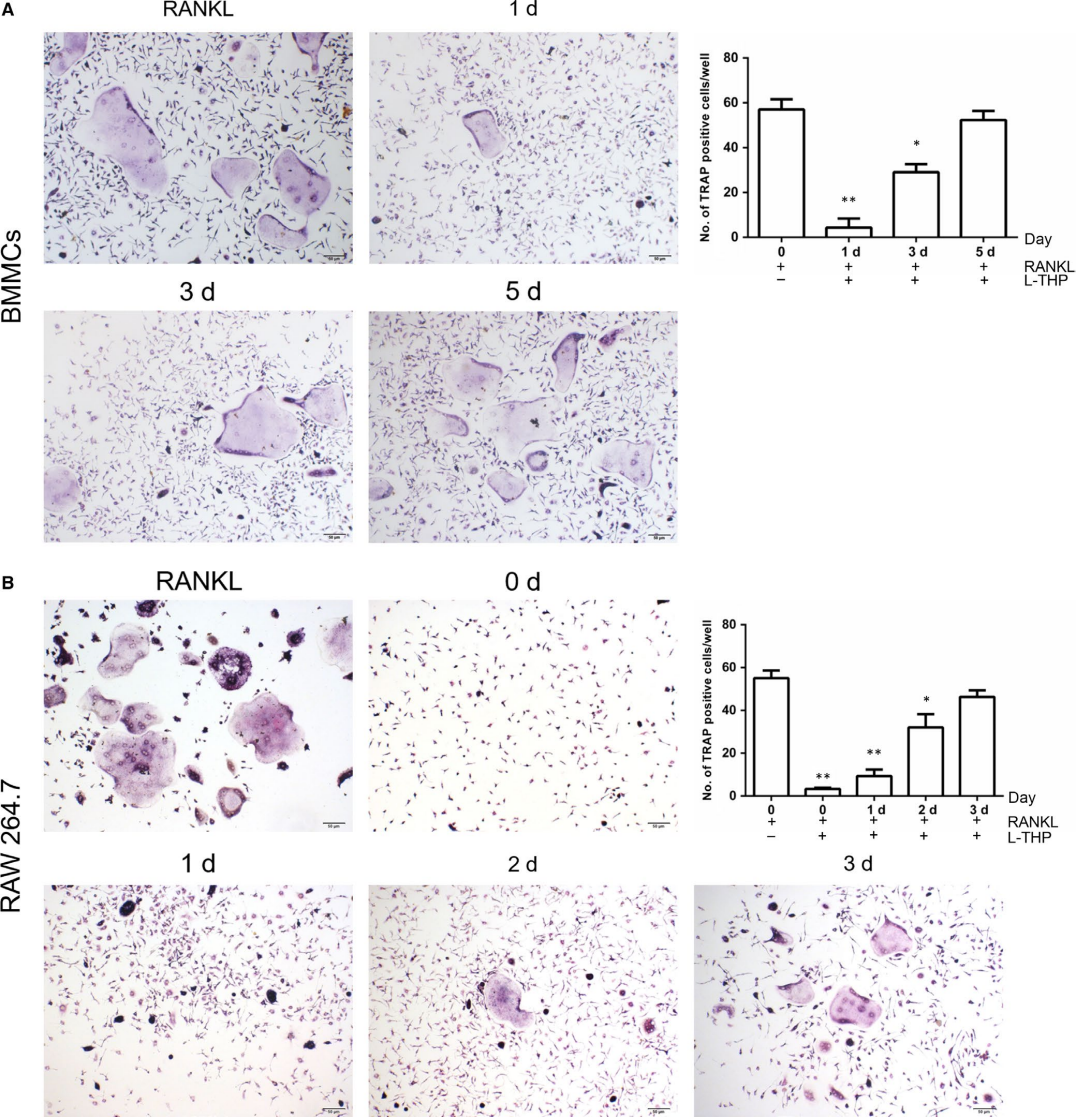
l‐tetrahydropalmatine (l‐THP) inhibits RANKL‐induced osteoclastogenesis at the early stage. A, BMMCs induced by M‐CSF (30 ng/mL) and RANKL (50 ng/mL) were treated with l‐THP (19 μg/mL) beginning on days 1, 3 and 5 respectively, with cell fixed and stained by TRAP on day 10. B, RAW 264.7 cells induced by M‐CSF (30 ng/mL) and RANKL (50 ng/mL) were treated with l‐THP (19 μg/mL) beginning on days 0, 1, 2 and 3 respectively, with cell fixed and stained by TRAP on day 10. (*P < .05, **P < .01)

To determine at which stage l‐THP treatment inhibited RANKL induced‐osteoclastogenesis, BMMCs were treated with l‐THP on days 1, 3 and 5, while RAW264.7 cells were treated on days 0, 1, 2 and 3. We observed that at the early stage, l‐THP inhibited osteoclast differentiation, but l‐THP was less effective at the later stage (Figure 3A‐B). Here, we concluded that l‐THP suppressed RANKL‐stimulated osteoclastogenesis at the early stage.

### 3.4 l‐THP inhibited activation of RANKL‐induced NF‐κB and MAPK pathways

**Figure 4:**
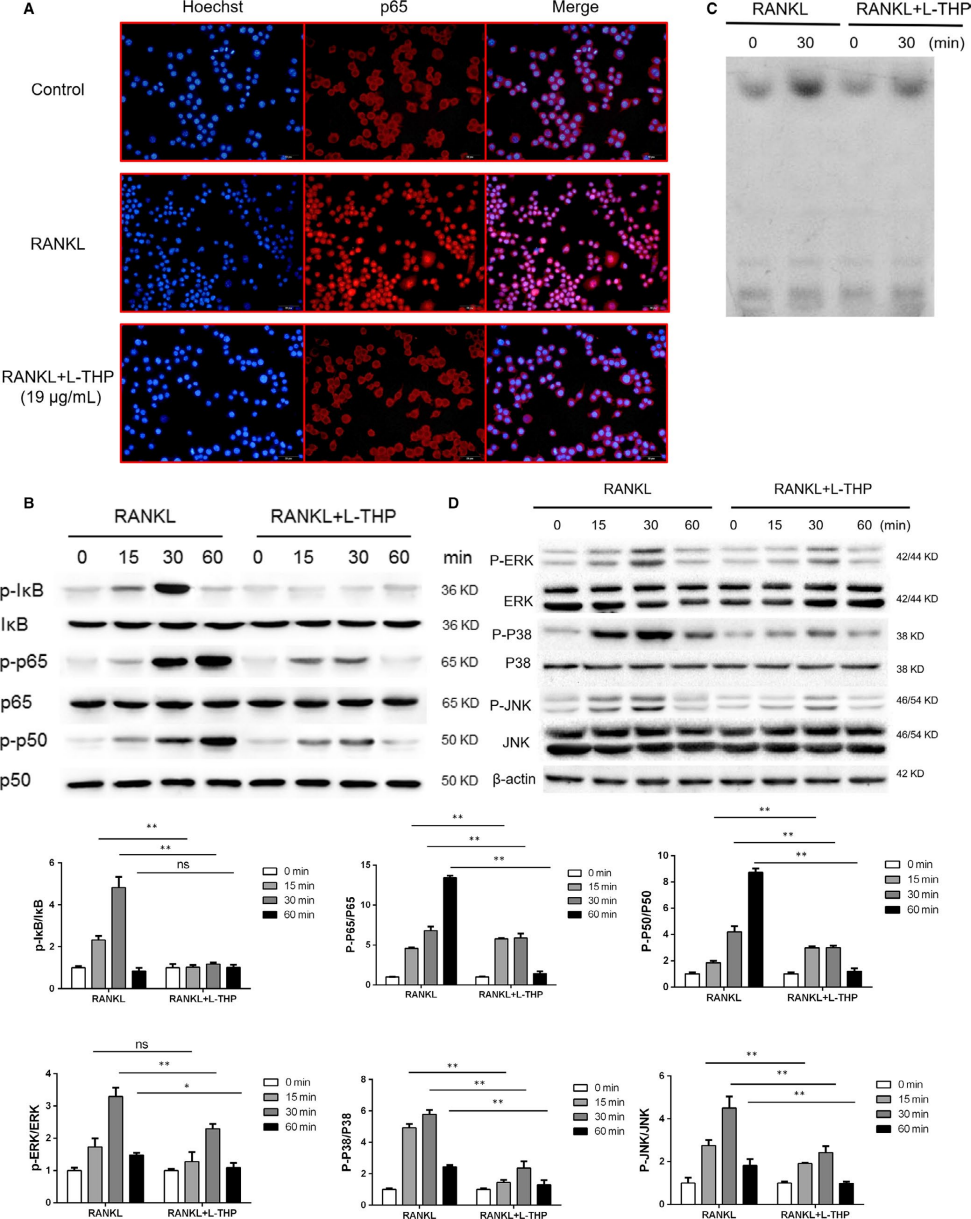
l‐tetrahydropalmatine (l‐THP) inhibits RANKL‐induced activation of NF‐κB and MAPK pathway. A, Induced RAW264.7 cells treated with l‐THP (19.00 μg/mL, 1h) were fixed with 40% formaldehyde, washed by Triton X‐100, followed by incubation with anti‐P65 antibody, goat antimouse IgG antibody and fluorescein‐conjugated streptavidin. B, Induced RAW264.7 cells treated with l‐THP (19.00 μg/mL) for 0, 15, 30 and 60 min. The levels of phosphorylated P65, P50 and IκB were evaluated by Western blotting analysis. C, Induced RAW264.7 cells treated with l‐THP (19.00 μg/mL) for 30 min, were then collected and resuspended with a 32P‐labelled DNA duplex and run in sodium dodecyl SDS‐PAGE system for EMSA analysis of the DNA binding activity of NF‐κB. D, Induced RAW 264.7 cells were treated with l‐THP (19.00 μg/mL) for 0, 15, 30 and 60 min, followed by Western blot analysis of the levels of phosphorylated ERK, JNK and P38. (*P < .05, **P < .01)

The NF‐κB pathway is also a critically important signalling pathway associated with RANKL‐mediated osteoclastogenesis. We used immunofluorescence staining of P65 to observe the nucleus translocation of P65. We observed that P65 was located in the cytoplasm of RAW264.7 cells without RANKL intervention, while P65 translocated to the nucleus after RANKL induction. However, l‐THP treatment reversed the effects of RANKL‐induction (Figure 4A). Western blotting revealed that l‐THP treatment inhibited the phosphorylation of P65, P50 and IκB (Figure 4B). We next performed an EMSA to determine the DNA binding activity of NF‐κB. The EMSA revealed that the DNA binding activity of NF‐κB was inhibited by the treatment of l‐THP (Figure 4C). MAPK subfamilies include P38, JNK and ERK, which phosphorylate after RANKL stimulation. Here, by using Western blotting, we evaluated the level of phosphorylation of factors of the MAPK pathway. The results revealed that the phosphorylation of P38, JNK and ERK significantly increased after the stimulation of RANKL. And the phosphorylation of ERK, JNK and P38 in osteoclastogenesis was decreased by l‐THP. It demonstrated that the activation of MAPK pathway also inhibited by l‐THP (Figure 4D). Taking together, these results indicated that l‐THP suppressed RANKL‐mediated activation of the NF‐κB and MAPK pathway in osteoclastogenesis.

### 3.5 l‐THP blocked RANK‐TRAF6 interactions

**Figure 5:**
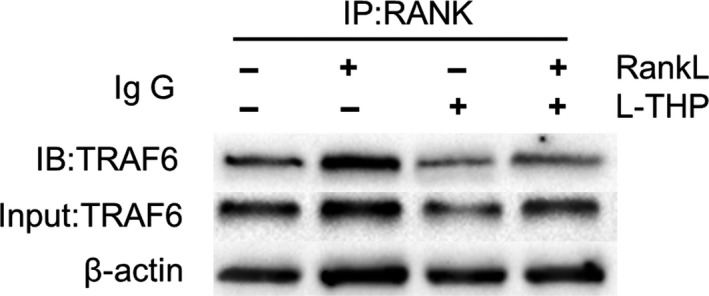
l‐tetrahydropalmatine (l‐THP) blocks RANK‐TRAF6 interactions. Induced RAW264.7 cells treated with l‐THP (19.00 μg/mL) for 60 min for CO‐IP analysis of RANK‐TRAF6 interaction

Recruitment of TRAF6 is critical to activating NF‐κB and MAPK pathway mediated by RANKL. To investigate whether l‐THP participates in TRAF6 recruitment after the activation of RANK, we conducted a Co‐IP experiment. RANKL‐RANK interaction recruited TRAF6 as our results confirmed. However, this effect was blocked by l‐THP treatment (Figure 5). The results indicated that treatment of l‐THP inhibited the recruitment and blocked the interactions of RANK‐TRAF6.

### 3.6 l‐THP inhibited the transcription of NFATc1 and overexpression of NFATc1 reversed the l‐THP effects on osteoclastogenesis

**Figure 6:**
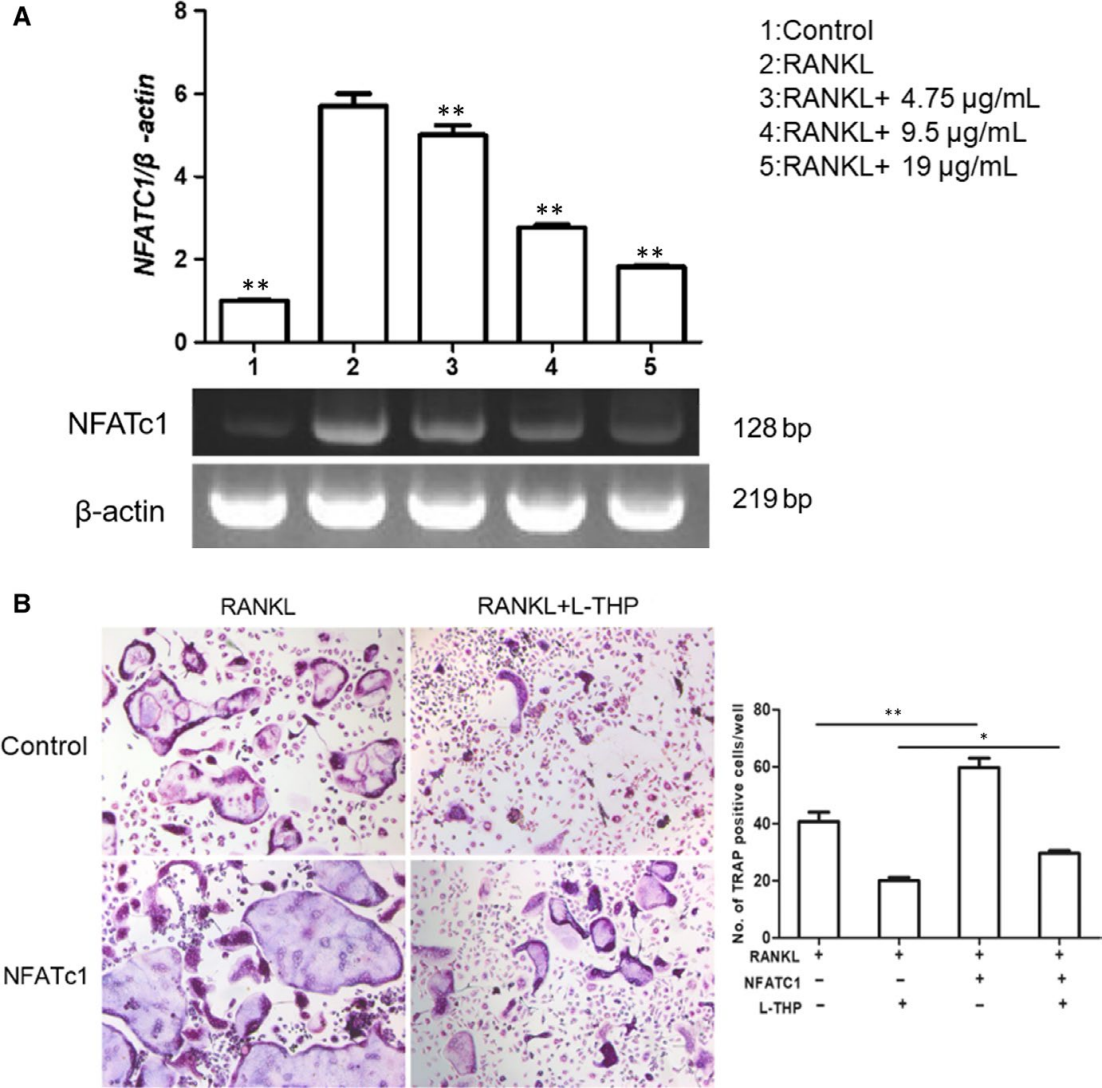
l‐tetrahydropalmatine (l‐THP) inhibits the activation of NFATc1 on osteoclastogenesis. A, RAW 264.7 cells were induced by M‐CSF (30 ng/mL) and RANKL (50 ng/mL), treated with l‐THP (0, 4.75, 9.5 and 19.00 μg/mL) for 7 days. Cells were collected for RT‐PCR analysis of NFATc1 mRNA level. B, Lentivirus vector was transfected into RAW 264.7 cells and after 3 days, and the transfected cells were induced and treated with l‐THP (19.00 μg/mL) for 7 days, then were fixed for TRAP staining (*P < .05, **P < .01)

To explore the relationship between l‐THP and the expression of NFATc1 after RANKL‐induction, we conducted an RT‐PCR assay. We observed that the mRNA level of NFATc1 significantly elevated after the incubation with RANKL. However, with the doses of l‐THP increased, the level of NFATc1 mRNA was decreased (Figure 6A). The results indicated that l‐THP dose‐dependently suppressed the mRNA transcription of NFATc1, which contributed to the inhibition of osteoclastogenesis.

To determine whether l‐THP exerted its effect depending on NFATc1 expression, we transfected Lv‐NFATc1 into RAW264.7 cells (Figure S1, MOI = 10) followed by incubation with RANKL to induce osteoclastogenesis. When NFATc1‐overexpressed RAW264.7 cells were stimulated into osteoclast differentiation, the number of mature osteoclasts was increased. l‐THP potently suppressed RANKL‐induced osteoclastogenesis, but the inhibitory effect was significantly reversed by overexpression of NFATc1 (Figure 6B). The results suggested that the inhibitory effect of l‐THP relied on the suppressive expression of NFATc1.

### 3.7 l‐THP prevented bone loss and inhibited osteoclastogenesis in ovariectomized mice

**Figure 7:**
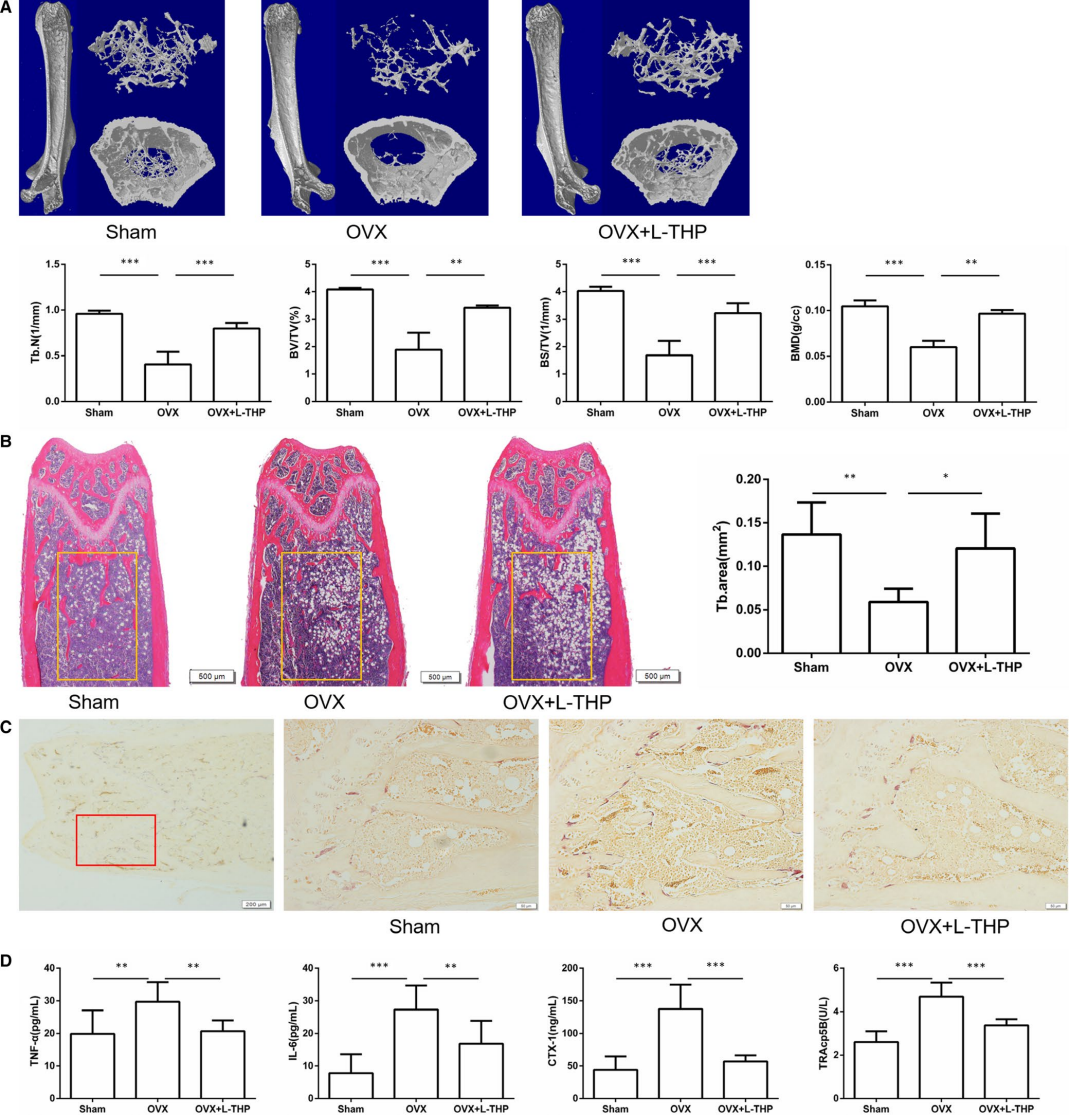
l‐tetrahydropalmatine (l‐THP) ameliorates ovariectomy‐induced bone loss in vivo. A, Micro CT analysis of the distal femurs from sham, OVX and OVX treated with l‐THP groups. B, Representative H&E staining of distal femoral sections and quantification of trabecular area. C, Representative TRAP‐stained distal femur sections from sham, OVX and OVX treated with l‐THP groups. D, Serum TNF‐α, IL‐6, CTX‐1 and TRAcp5B were examined (*P < .05, **P < .01, ***P < .001)

To examine the effect of l‐THP on bone loss and osteoclastogenesis in vivo, we performed the in vivo experiment by using an OVX model. As the results showed, 6 weeks after the surgery, OVX mice exhibited decreased trabecular numbers, BMD, trabecular BS/TV, trabecular BV/TV, when compared with the sham group, as demonstrated by micro CT. Treatment with l‐THP prevented the decline of bone mass (Figure 7A). These findings were similar to the results of H&E staining as showed in Figure 7B.

We next conducted TRAP staining experiments to determine whether l‐THP inhibited osteoclastogenesis in vivo. l‐THP markedly reduced the TRAP‐positive cells in the femur (Figure 7C). And serum TNF‐α, IL‐6, CTX‐1 and TRAcp5B levels in l‐THP ‐treated group were significantly lower than those in the OVX group (Figure 7D).

### 3.8 l‐THP showed little effect on osteogenesis and adipogenesis

To identify the role of l‐THP on osteogenesis and adipogenesis, we conducted ALP, Alizarin Red and Oil Red O staining. After osteogenic induction, with or without l‐THP treatment, there presented no significant difference not only in the number of ALP‐positive cells but also in the calcium nodules formation (Figure S2). Oil Red O staining also showed that the level of fat granules formation was not inhibited by l‐THP (Figure S3). Taking together, these results showed that l‐THP exhibited no effects on osteogenesis and adipogenesis.

### 3.9 l‐THP showed little effect on C‐Fms expression and inhibited RANK expression during osteoclastogenesis

At the process of osteoclastogenesis, BMMCs differentiate into osteoclast precursors, which further differentiate into osteoclasts stimulated by RANKL at the later stage. To exclude the probable effects of l‐THP on RANK and C‐Fms induced by M‐CSF, we conducted RT‐PCR to evaluate the mRNA level of RANK and C‐Fms. The mRNA level of RANK increased by M‐CSF induction and l‐THP inhibited the transcription of RANK. C‐Fms was increased by M‐CSF and l‐THP showed no effect on transcription of C‐Fms (Figure S4).

## 4 DISCUSSION

In this study, we demonstrated that l‐THP significantly inhibited osteoclastogenesis in vitro and OVX‐induced bone loss in vivo. As for the molecular mechanism, l‐THP blocked the RANK‐TRAF6 interactions and further suppressed the activation of NF‐κB and MAPK pathways during osteoclastogenesis. As a result, the master transcription of NFAFc1 was down‐regulated and the expression of osteoclast‐related marker genes followed. To the best of our knowledge, it is the first report of l‐THP’s protective effect on metabolic bone disorders. The solid inhibitory effects of l‐THP against osteoclastogenesis make it a promising natural agent for the treatment of osteoporosis and other overactivated bone resorptive diseases.

l‐tetrahydropalmatine, an alkaloid extracted from Corydalis, has shown its effects on protecting endothelial cells against inflammation, alleviating myocardial ischaemia‐reperfusion injury.^25^, ^26^, ^27^ Another research also indicated that l‐THP inhibited inflammation and protected the rat against D–galactose induced memory impairment through the inhibition of NF‐κB pathway.^17^ It is well known that the imbalance of bone metabolism is caused by the interrupted bone formation and excessive bone resorption, which was initialed by the micro‐inflammatory status by oestrogen withdrawal.^28^, ^29^ Therefore, whether l‐THP exerts protective effects against pathological bone loss such as post‐menopausal osteoporosis, is quite interesting and meaningful, yet remains unclear. As a result, we conduct this experiment in an effort to determine the effects of l‐THP on osteoclastogenesis and oestrogen‐deficiency diseases mimicked by an ovariectomized mice model.

Before we started, we performed MTT assay and selected three concentrations (4.75, 9.5 and 19 μg/mL) with little cytotoxic effects for the following in vitro experiments. The delicate F‐actin ring structure is crucial for matured osteoclasts to digest and resorb bone matrix.^30^ Hence, we performed F‐actin ring formation assay and bone resorption assay to investigate whether l‐THP impaired bone resorption activity. The results showed that both the F‐actin ring formation and bone resorption were disrupted by l‐THP in a dose‐dependent manner, which cohered with the dose‐dependent inhibition of osteoclastogenesis demonstrated by TRAP staining. Also, the expression of TRAP, Cathepsin K, CTR and MMP‐9 was all indicators of osteoclastogenesis and osteoclastic activity. As such, we examined the expression of osteoclastogenesis‐related marker genes, and we found l‐THP significantly suppressed the mRNA transcription activity of the above genes. In addition, we determined l‐THP inhibited osteoclastogenesis at the early stage rather than the later stage.

Oestrogen deficiency also plays a detrimental role in determining the commitment of BMSCs differentiation, which renders BMSCs in favour of adipogenesis instead of osteogenesis.^31^ Here in our report, we found that l‐THP showed little effects on the osteogenesis and adipogenesis of BMSCs, demonstrated by ALP and Alizarin red staining, as well as Oil red staining.

Two indispensable receptors, the receptor of RANKL and M‐CSF, namely RANK and c‐Fms, dominate two different stages of the osteoclastogenesis process, which include the proliferation stage by M‐CSF binding to c‐Fms and the differentiation stage by RANKL binding to RANK.^32^, ^33^ In our study, RT‐PCR results showed that l‐THP inhibited the transcription of RANK but showed limited effect on c‐Fms transcription.

During osteoclastogenesis, after the combination of RANKL and RANK, TRAF6 was recruited by RANK to further activate the phosphorylation of P65, IκB, P50 (NF‐κB pathway), ERK, JNK and P38 (MAPK pathway).^34^, ^35^ In this study, we found that l‐THP significantly inhibited the recruitment of TRAF6 after RANK was activated by RANKL and suppressed the phosphorylation of IκB, P65 and P50 in NF‐κB pathway and P38, JNK and ERK in MAPK pathway. Besides, the immunofluorescence staining results showed a dampened nuclear translocation of P65. The inhibition of NF‐κB signalling of l‐THP demonstrated the consistency of the previous finding.^17^

As the downstream target of NF‐κB and MAPK pathways, NFATc1 was the most important transcription factor for osteoclastogenesis.^36^, ^37^, ^38^ The activation of NFATc1 triggers the expression of osteoclastogenesis‐related marker genes, such as TRAP, Cathepsin K, CTR and MMP‐9. Here, we showed that l‐THP inhibited the transcription of NFATc1 in the process of osteoclastogenesis. Notably, after the overexpression of NFATc1 in RAW264.7 cells, the inhibitory effects of l‐THP on osteoclastogenesis were partially reversed, suggesting that l‐THP took effect mainly in the upstream of the RANKL signalling pathway. Overall, l‐THP blocked the interactions of RANK‐TRAF6 and further inhibited the activation of NF‐κB and MAPK pathways which resulted in the decrease in NFATc1 expression, ultimately suppressing osteoclastogenesis.

Bone homeostasis is delicately maintained by the balance of osteoblasts and osteoclasts.^18^, ^19^, ^20^ The imbalance of bone metabolism in PMOP is associated with oestrogen withdrawal.^39^ Oestrogen withdrawal could lead to the activation of inflammation which facilitates the overactivation of osteoclasts and further leads to the increased bone turnover rate resulting in bone loss.^40^ Inhibiting osteoclastogenesis is still an effective strategy to alleviate PMOP and other osteoclast‐related disorders.^21^, ^22^, ^23^, ^24^ Therefore, we explored the effects of l‐THP on OVX‐induced bone loss and the potential mechanism. In vivo, HE staining and micro CT analysis showed that l‐THP significantly inhibited the bone loss induced by OVX. TRAP staining indicated that the number of TRAP‐positive cells (osteoclasts) surrounding the trabecula was significantly decreased after treated with l‐THP. Meanwhile, l‐THP reduced the serum CTX‐1 and TRAcp5B levels. Collectively, these data suggested that l‐THP inhibited OVX‐induced bone loss and suppressed osteoclastogenesis in vivo.

Bisphosphonates were widely used osteoclast suppressors in the treatment of osteoporosis. However, osteonecrosis of the jaws causes major concerns of the clinical use of bisphosphonates.^41^ Hence, exploring more efficient and safer osteoclastogenesis specific inhibitor benefits the medical treatment of osteoporosis. In our report, l‐THP was found to be safe without severe complication in our in vivo experiment and would be a promising natural agent treating osteoporosis.

However, there are still some limitations of this study. Firstly, l‐THP is used safely to treat insomnia and relieve pain in China for decades^42^; however, the effects of l‐THP on bone metabolism of human‐being are rarely examined. Therefore, it would be interesting to further explore the treatment efficacy and the side effects of l‐THP in patients with osteoclast‐related disorders and compare the results with other drugs or treatments. Secondly, we proved that l‐THP could inhibit osteoclastogenesis by the blockage of RANK‐TRAF6 interactions and inhibition of NF‐κB and MAPK pathways but we did not identify the exact target that l‐THP acted on, which needs many further works on it.

Collectively, our study demonstrated that l‐THP inhibited OVX‐induced bone loss in vivo and suppressed osteoclastogenesis by the blockage of RANK‐TRAF6 interactions and inhibition of NF‐κB and MAPK pathways in vitro and in vivo. This evidence will make l‐THP a promising candidate for the treatment of PMOP and other osteoclastogenesis‐related bone diseases.

## CONFLICT OF INTEREST

The authors declare no conflict of interests.

## AUTHOR CONTRIBUTIONS

XZ, XC and JS designed the study. XZ, JC, YH, XL, HJ, YJW and LW involved in data collection. HC, LC, YW, QZ, SW and LQ carried out the data analysis. XC and JS interpreted the data. XZ, LW, WW, HS and CF drafted the manuscript. XZ, XC and JS revised the manuscript. SJ approved final version of manuscript.

## Data Availability

Data used to support the findings of this study has been presented in the Supplementary Information.
