# Supplementary figures and images for: l‐tetrahydropalmatine suppresses osteoclastogenesis in vivo and in vitro via blocking RANK‐TRAF6 interactions and inhibiting NF‐κB and MAPK pathways
Source: J Cell Mol Med. 2019 Nov 14;24(1):785–98. doi: 10.1111/jcmm.14790 (PMC6933417; doi:10.1111/jcmm.14790)

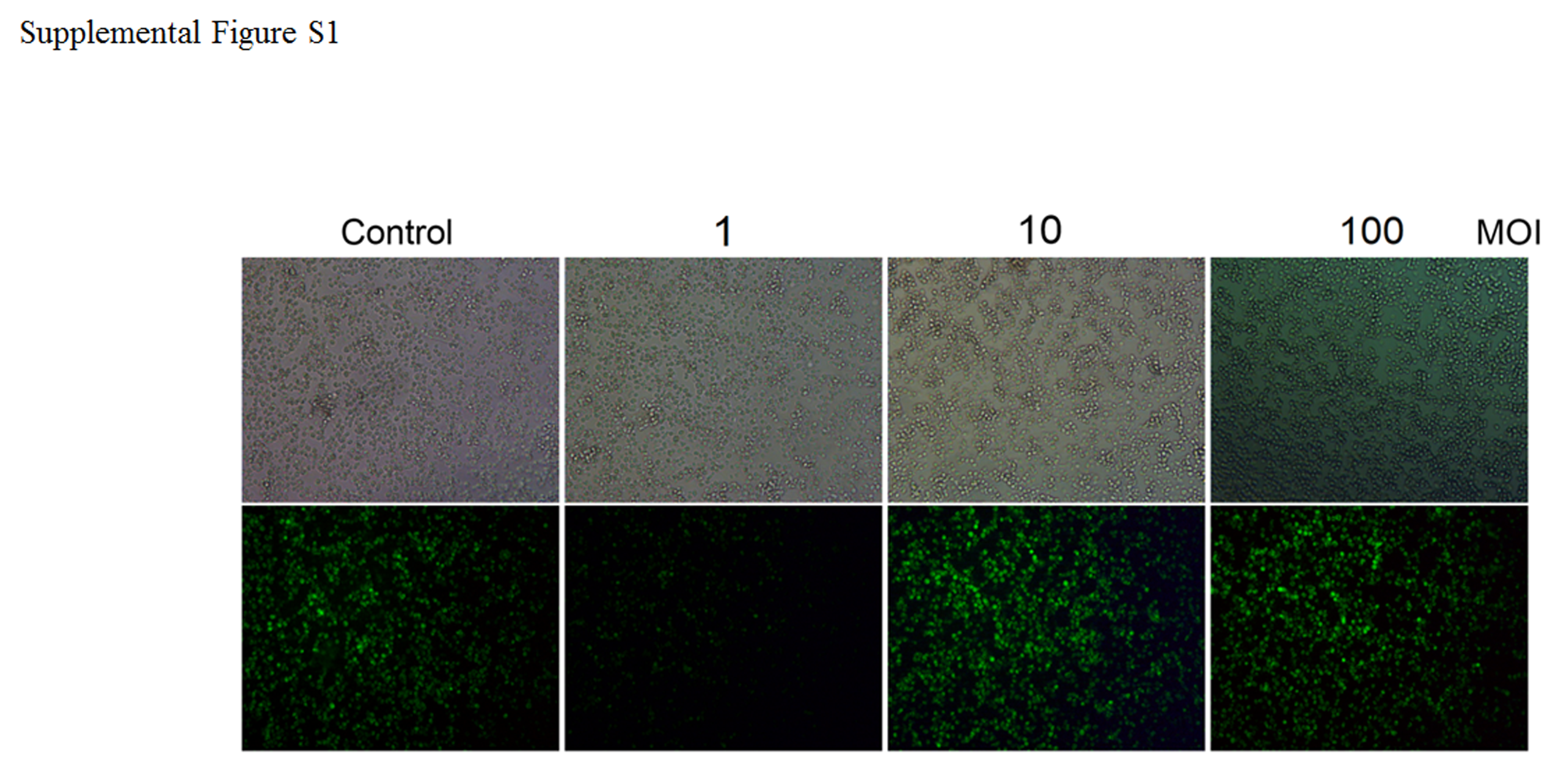

Supplement: Supplementary file 1 [file JCMM-24-785-s001.tif]

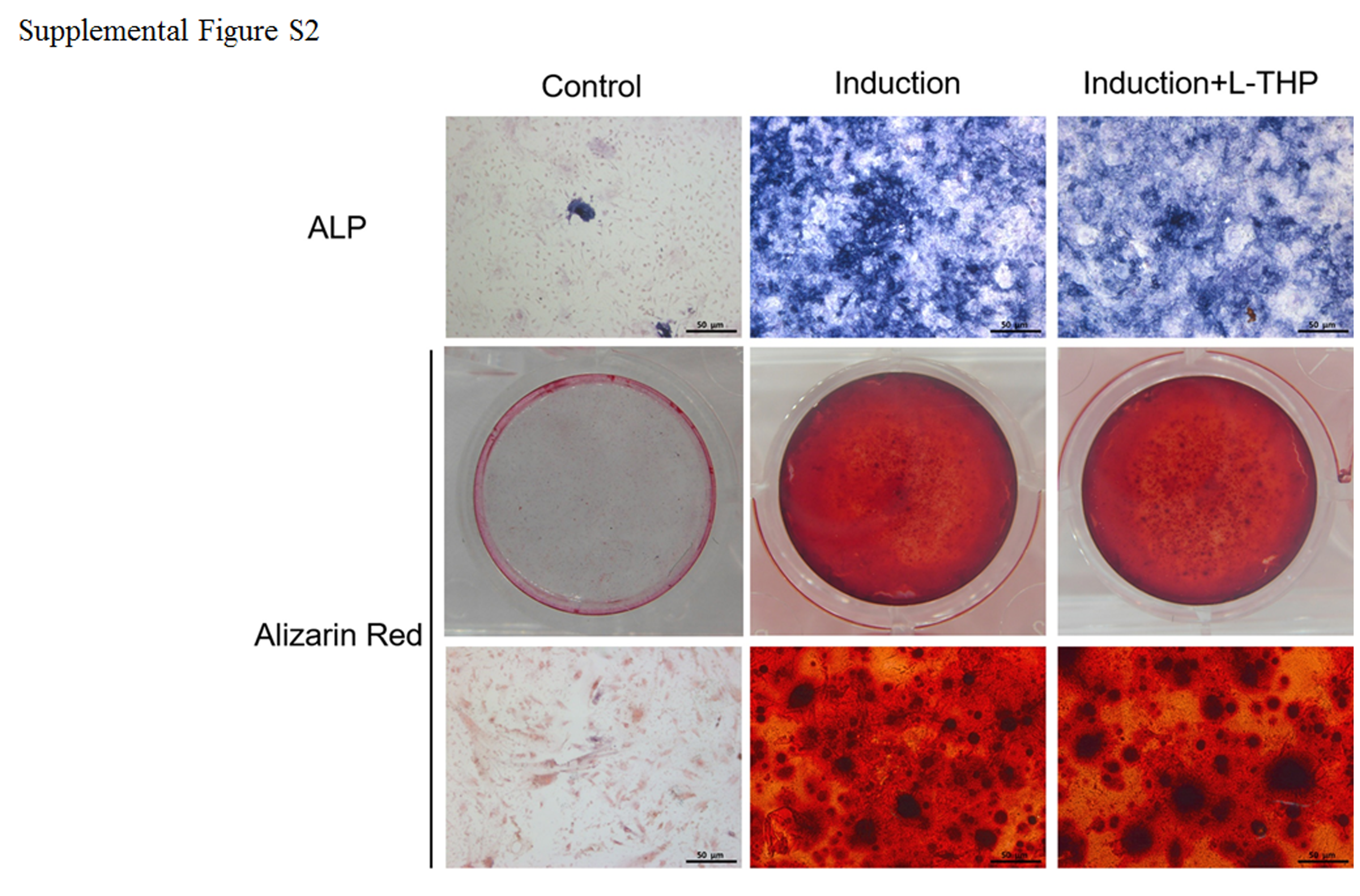

Supplement: Supplementary file 2 [file JCMM-24-785-s002.tif]

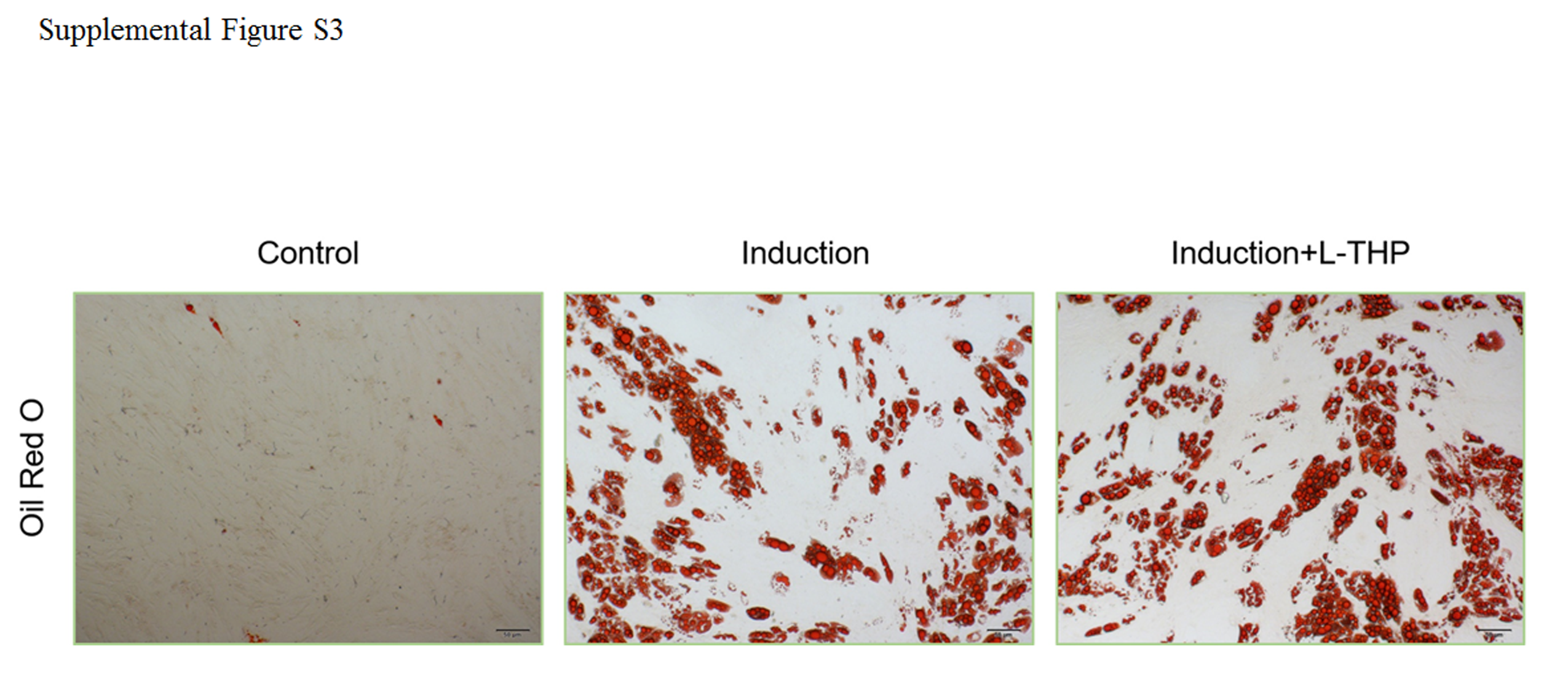

Supplement: Supplementary file 3 [file JCMM-24-785-s003.tif]

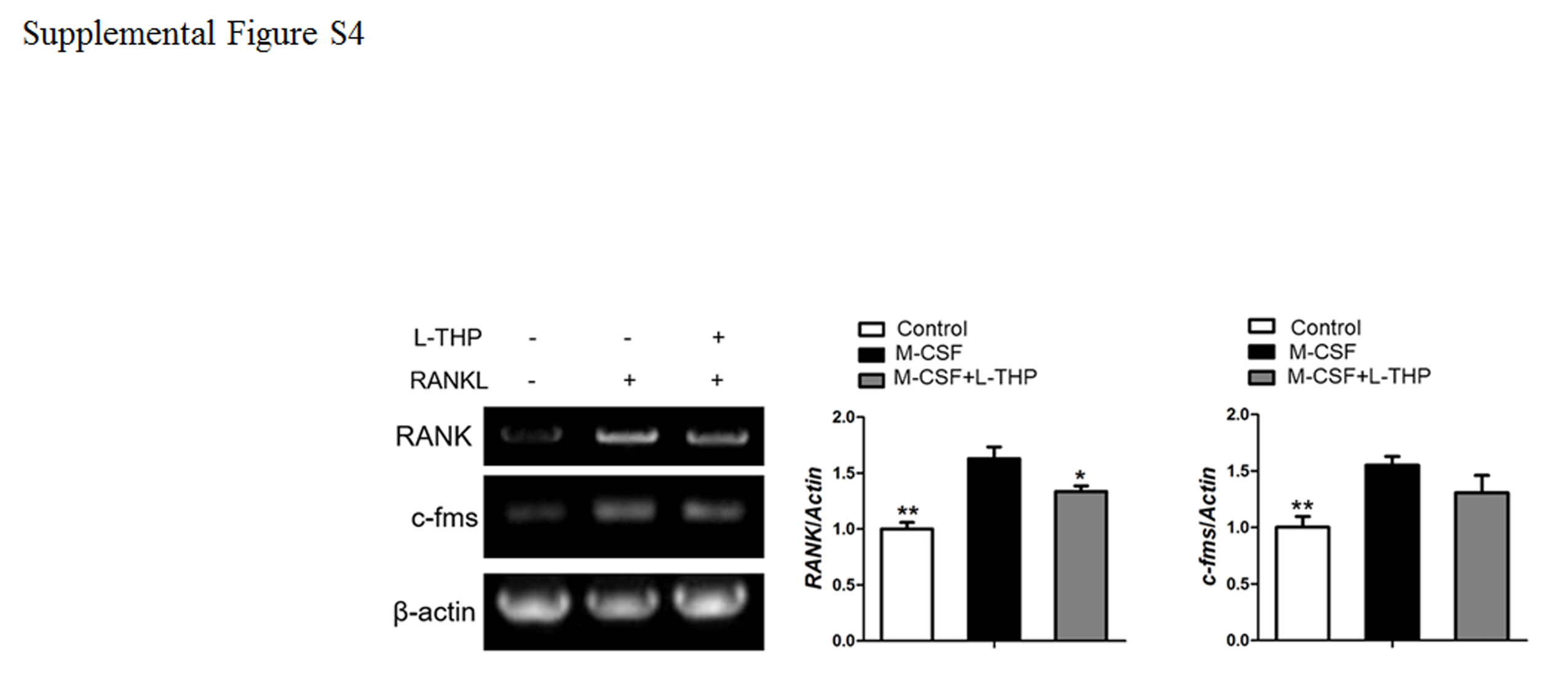

Supplement: Supplementary file 4 [file JCMM-24-785-s004.tif]
