# Supplementary material for: l‐tetrahydropalmatine suppresses osteoclastogenesis in vivo and in vitro via blocking RANK‐TRAF6 interactions and inhibiting NF‐κB and MAPK pathways
Source: J Cell Mol Med. 2019 Nov 14;24(1):785–98. doi: 10.1111/jcmm.14790 (PMC6933417; doi:10.1111/jcmm.14790)
Supplement: Supplementary file 5 [file JCMM-24-785-s005.doc]

**Figure 1**B

| L-THP Concentration (μg/ml) | Mean | SD |
| --- | --- | --- |
| 0 | 100.00 | 0.21 |
| 2.36 | 99.73 | 0.16 |
| 4.73 | 99.10 | 0.32 |
| 9.47 | 98.20 | 0.45 |
| 18.95 | 98.74 | 0.43 |
| 37.92 | 75.110 | 1.96 |
| 75.83 | 64.50 | 0.09 |

**Figure 1C**

| Group | Mean | SD | P value |
| --- | --- | --- | --- |
| 0 μg/ml | 1.33 | 0.58 | / |
| 0 μg/ml+RANKL | 56.67 | 4.51 | / |
| 4.75 μg/ml+RANKL | 44.67 | 3.79 | / |
| 9.5 μg/ml +RANKL | 27.67 | 3.51 | / |
| 19 μg/ml +RANKL | 6.667 | 3.79 | / |
| 0 μg/ml+RANKL VS 0 μg/ml | / | / | <0.001 |
| 0 μg/ml+RANKL VS 4.75 μg/ml+RANKL | / | / | ＜0.05 |
| 0 μg/ml+RANKL VS 9.5 μg/ml+RANKL | / | / | ＜0.01 |
| 0 μg/ml+RANKL VS 19 μg/ml+RANKL | / | / | ＜0.001 |

**Figure 1D**

| Group | Mean | SD | P value |
| --- | --- | --- | --- |
| 0 μg/ml | 1.28 | 0.46 | / |
| 0 μg/ml+RANKL | 57.00 | 4.00 | / |
| 4.75 μg/ml+RANKL | 44.33 | 3.21 | / |
| 9.5 μg/ml +RANKL | 35.67 | 5.69 | / |
| 19 μg/ml +RANKL | 8.00 | 2.65 | / |
| 0 μg/ml+RANKL VS 0 μg/ml | / | / | <0.001 |
| 0 μg/ml+RANKL VS 4.75 μg/ml+RANKL | / | / | <0.05 |
| 0 μg/ml+RANKL VS 9.5 μg/ml+RANKL | / | / | <0.01 |
| 0 μg/ml+RANKL VS 19 μg/ml+RANKL | / | / | <0.01 |

**Figure 2A**

| Group | Mean | SD | P value |
| --- | --- | --- | --- |
| 0 μg/ml | 1.00 | 0.00 | / |
| 0 μg/ml+RANKL | 47.00 | 3.00 | / |
| 4.75 μg/ml+RANKL | 34.33 | 2.52 | / |
| 9.5 μg/ml +RANKL | 19.67 | 2.52 | / |
| 19 μg/ml +RANKL | 9.00 | 1.00 | / |
| 0 μg/ml+RANKL VS 0 μg/ml | / | / | <0.01 |
| 0 μg/ml+RANKL VS 4.75 μg/ml+RANKL | / | / | <0.05 |
| 0 μg/ml+RANKL VS 9.5 μg/ml+RANKL | / | / | <0.01 |
| 0 μg/ml+RANKL VS 19 μg/ml+RANKL | / | / | <0.01 |

**Figure 2B**

| Group | Mean | SD | P value |
| --- | --- | --- | --- |
| 0 μg/ml | 0.00 | 0.00 | / |
| 0 μg/ml+RANKL | 0.42 | 0.03 | / |
| 4.75 μg/ml+RANKL | 0.19 | 0.02 | / |
| 9.5 μg/ml +RANKL | 0.09 | 0.01 | / |
| 19 μg/ml +RANKL | 0.05 | 0.01 | / |
| 0 μg/ml+RANKL VS 0 μg/ml | / | / | <0.01 |
| 0 μg/ml+RANKL VS 4.75 μg/ml+RANKL | / | / | <0.01 |
| 0 μg/ml+RANKL VS 9.5 μg/ml+RANKL | / | / | <0.01 |
| 0 μg/ml+RANKL VS 19 μg/ml+RANKL | / | / | <0.01 |

**Figure 2C** (Cathepsin K/β-actin)

| Group | Mean | SD | P value |
| --- | --- | --- | --- |
| 0 μg/ml | 1.00 | 0.084 | / |
| 0 μg/ml+RANKL | 3.52 | 0.12 | / |
| 4.75 μg/ml+RANKL | 2.97 | 0.06 | / |
| 9.5 μg/ml +RANKL | 2.42 | 0.059 | / |
| 19 μg/ml +RANKL | 1.91 | 0.15 | / |
| 0 μg/ml+RANKL VS 0 μg/ml | / | / | <0.01 |
| 0 μg/ml+RANKL VS 4.75 μg/ml+RANKL | / | / | <0.01 |
| 0 μg/ml+RANKL VS 9.5 μg/ml+RANKL | / | / | <0.01 |
| 0 μg/ml+RANKL VS 19 μg/ml+RANKL | / | / | <0.01 |

**Figure 2C** (TRAP/β-actin)

| Group | Mean | SD | P value |
| --- | --- | --- | --- |
| 0 μg/ml | 1.00 | 0.03 | / |
| 0 μg/ml+RANKL | 3.47 | 0.10 | / |
| 4.75 μg/ml+RANKL | 2.96 | 0.08 | / |
| 9.5 μg/ml +RANKL | 2.39 | 0.16 | / |
| 19 μg/ml +RANKL | 1.87 | 0.09 | / |
| 0 μg/ml+RANKL VS 0 μg/ml | / | / | <0.01 |
| 0 μg/ml+RANKL VS 4.75 μg/ml+RANKL | / | / | <0.01 |
| 0 μg/ml+RANKL VS 9.5 μg/ml+RANKL | / | / | <0.01 |
| 0 μg/ml+RANKL VS 19 μg/ml+RANKL | / | / | <0.01 |

**Figure 2C** (CTR/β-actin)

| Group | Mean | SD | P value |
| --- | --- | --- | --- |
| 0 μg/ml | 1.00 | 0.09 | / |
| 0 μg/ml+RANKL | 2.44 | 0.17 | / |
| 4.75 μg/ml+RANKL | 1.65 | 0.01 | / |
| 9.5 μg/ml +RANKL | 1.36 | 0.06 | / |
| 19 μg/ml +RANKL | 1.14 | 0.11 | / |
| 0 μg/ml+RANKL VS 0 μg/ml | / | / | <0.01 |
| 0 μg/ml+RANKL VS 4.75 μg/ml+RANKL | / | / | <0.01 |
| 0 μg/ml+RANKL VS 9.5 μg/ml+RANKL | / | / | <0.01 |
| 0 μg/ml+RANKL VS 19 μg/ml+RANKL | / | / | <0.01 |

**Figure 2C** (MMP-9/β-actin)

| Group | Mean | SD | P value |
| --- | --- | --- | --- |
| 0 μg/ml | 1.00 | 0.11 | / |
| 0 μg/ml+RANKL | 4.14 | 0.17 | / |
| 4.75 μg/ml+RANKL | 3.43 | 0.12 | / |
| 9.5 μg/ml +RANKL | 2.02 | 0.26 | / |
| 19 μg/ml +RANKL | 1.54 | 0.04 | / |
| 0 μg/ml+RANKL VS 0 μg/ml | / | / | <0.01 |
| 0 μg/ml+RANKL VS 4.75 μg/ml+RANKL | / | / | <0.01 |
| 0 μg/ml+RANKL VS 9.5 μg/ml+RANKL | / | / | <0.01 |
| 0 μg/ml+RANKL VS 19 μg/ml+RANKL | / | / | <0.01 |

**Figure 3A**

| Group | Mean | SD | P value |
| --- | --- | --- | --- |
| 0 d+RANKL | 58.00 | 4.00 | / |
| 1 d+L-THP+RANKL | 5.33 | 2.08 | / |
| 3 d+L-THP+RANKL | 29.67 | 2.52 | / |
| 5 d+ L-THP+RANKL | 49.33 | 3.06 | / |
| 0 d +RANKL VS 1 d+L-THP+RANKL | / | / | <0.01 |
| 0 d +RANKL VS 3 d+L-THP+RANKL | / | / | <0.05 |
| 0 d +RANKL VS 5 d+L-THP+RANKL | / | / | >0.05 |

**Figure 3B**

| Group | Mean | SD | P value |
| --- | --- | --- | --- |
| 0 d+RANKL | 56.00 | 4.00 | / |
| 0 d+L-THP+RANKL | 3.67 | 1.53 |  |
| 1 d+L-THP+RANKL | 10.33 | 2.52 | / |
| 2 d+L-THP+RANKL | 33.00 | 4.59 | / |
| 3 d+ L-THP+RANKL | 47.33 | 3.06 | / |
| 0 d +RANKL VS 0 d+L-THP+RANKL | / | / | <0.01 |
| 0 d +RANKL VS 1 d+L-THP+RANKL | / | / | <0.01 |
| 0 d +RANKL VS 2 d+L-THP+RANKL | / | / | <0.05 |
| 0 d +RANKL VS 3 d+L-THP+RANKL | / | / | >0.05 |

**Figure 4B**

| Group | Mean Difference | SD Difference | P value |
| --- | --- | --- | --- |
| (p-IκB/ IκB RANKL-15min /  p-IκB/ IκB RANKL 0min) vs. (p-IκB/ IκB RANKL+L-THP-15min /  p-IκB/ IκB RANKL+L-THP 0min) | 1.305 | 0.1782 | <0.01 |
| (p-IκB/ IκB RANKL-30min /  p-IκB/ IκB RANKL 0min) vs. (p-IκB/ IκB RANKL+L-THP-30min /  p-IκB/ IκB RANKL+L-THP 0min) | 3.645 | 0.1782 | <0.01 |
| (p-IκB/ IκB RANKL-60min /  p-IκB/ IκB RANKL 0min) vs. (p-IκB/ IκB RANKL+L-THP-60min /  p-IκB/ IκB RANKL+L-THP 0min) | -0.1676 | 0.1782 | >0.05 |
| (p-P65/ P65 RANKL-15min /  p-P65/ P65 RANKL 0min) vs. (p-P65/ P65 RANKL+L-THP-15min /  p-P65/ P65 RANKL+L-THP 0min) | -1.198 | 0.2466 | <0.01 |
| (p-P65/ P65 RANKL-30min /  p-P65/ P65 RANKL 0min) vs. p-P65/ P65 RANKL+L-THP-30min /  p-P65/ P65 RANKL+L-THP 0min) | 0.9389 | 0.2466 | <0.01 |
| (p-P65/ P65 RANKL-60min /  p-P65/ P65 RANKL 0min) vs. (p-P65/ P65 RANKL+L-THP-60min /  p-P65/ P65 RANKL+L-THP 0min) | 12.01 | 0.2466 | <0.01 |
| (p-P50/ P50 RANKL-15min /  p-P50/ P50 RANKL 0min) vs. (p-P50/ P50 RANKL+L-THP-15min /  p-P50/ P50 RANKL+L-THP 0min) | -1.127 | 0.1879 | <0.01 |
| (p-P50/ P50 RANKL-30min /  p-P50/ P50 RANKL 0min) vs. (p-P50/ P50 RANKL+L-THP-30min /  p-P50/ P50 RANKL+L-THP 0min) | 1.197 | 0.1879 | <0.01 |
| (p-P50/ P50 RANKL-60min /  p-P50/ P50 RANKL 0min) vs. (p-P50/ P50 RANKL+L-THP-60min /  p-P50/ P50 RANKL+L-THP 0min) | 7.523 | 0.1879 | <0.01 |

**Figure 4D**

| Group | Mean Difference | SD Difference | P |
| --- | --- | --- | --- |
| (p-ERK/ ERK RANKL-15min /  p-ERK/ ERK RANKL 0min) vs. (p-ERK/ ERK RANKL+L-THP-15min /  p-ERK/ ERK RANKL+L-THP 0min) | 0.4534 | 0.1557 | >0.05 |
| (p-ERK/ ERK RANKL-30min /  p-ERK/ ERK RANKL 0min) vs. (p-ERK/ ERK RANKL+L-THP-30min /  p-ERK/ ERK RANKL+L-THP 0min) | 1.000 | 0.1557 | <0.01 |
| (p-ERK/ ERK RANKL-60min /  p-ERK/ ERK RANKL 0min) vs. (p-ERK/ ERK RANKL+L-THP-60min /  p-ERK/ ERK RANKL+L-THP 0min) | 0.3876 | 0.1557 | <0.05 |
| (p-P38/ P38 RANKL-15min /  p-P38/ P38 RANKL 0min) vs. (p-P38/ P38 RANKL+L-THP-15min /  p-P38/ P38 RANKL+L-THP 0min) | 3.487 | 0.1925 | <0.01 |
| (p-P38/ P38 RANKL-30min /  p-P38/ P38 RANKL 0min) vs. p-P38/ P38 RANKL+L-THP-30min /  p-P38/ P38 RANKL+L-THP 0min) | 3.411 | 0.1925 | <0.01 |
| (p-P38/ P38 RANKL-60min /  p-P38/ P38 RANKL 0min) vs. (p-P38/ P38 RANKL+L-THP-60min /  p-P38/ P38 RANKL+L-THP 0min) | 1.149 | 0.1925 | <0.01 |
| (p-JNK/ JNK RANKL-15min /  p-JNK/ JNK RANKL 0min) vs. (p-JNK/ JNK RANKL+L-THP-15min /  p-JNK/ JNK RANKL+L-THP 0min) | 0.8402 | 0.2250 | <0.01 |
| (p-JNK/ JNK RANKL-30min /  p-JNK/ JNK RANKL 0min) vs. (p-JNK/ JNK RANKL+L-THP-30min /  p-JNK/ JNK RANKL+L-THP 0min) | 2.073 | 0.2250 | <0.01 |
| (p-JNK/ JNK RANKL-60min /  p-JNK/ JNK RANKL 0min) vs. (p-JNK/ JNK RANKL+L-THP-60min /  p-JNK/ JNK RANKL+L-THP 0min) | 0.8376 | 0.2250 | <0.01 |

**Figure 6A** (NFATc1/β-actin)

| Group | Mean | SD | P value |
| --- | --- | --- | --- |
| 0 μg/ml | 1 | 0.02 | / |
| 0 μg/ml+RANKL | 5.71 | 0.50 | / |
| 4.75 μg/ml+RANKL | 5.00 | 0.41 | / |
| 9.5 μg/ml +RANKL | 2.77 | 0.12 | / |
| 19 μg/ml +RANKL | 1.82 | 0.05 | / |
| 0 μg/ml+RANKL VS 0 μg/ml | / | / | <0.01 |
| 0 μg/ml+RANKL VS 4.75 μg/ml+RANKL | / | / | <0.01 |
| 0 μg/ml+RANKL VS 9.5 μg/ml+RANKL | / | / | <0.01 |
| 0 μg/ml+RANKL VS 19 μg/ml+RANKL | / | / | <0.01 |

**Figure 6B**

| Group | Mean | SD | P value |
| --- | --- | --- | --- |
| RANKL | 40.33 | 3.51 | / |
| RANKL+L-THP | 19.33 | 1.15 | / |
| RANKL+NFATc1 | 59.67 | 3.51 | / |
| RANKL+L-THP+NFATc1 | 29.33 | 1.15 | / |
| RANKL VS RANKL+NFATc1 | / | / | <0.01 |
| RANKL+L-THP VS RANKL+L-THP+NFATc1 | / | / | <0.05 |

**Figure 7A** (Tb.N)

| Group | Mean | SD | P value |
| --- | --- | --- | --- |
| Sham | 0.9586 | 0.0344 | Sham VS OVX: 0.000 |
| OVX | 0.4061 | 0.1379 |  |
| OVX+L-THP | 0.7981 | 0.0613 | OVX VS OVX+L-THP: 0.000 |

**Figure 7A (BV/TV)**

| Group | Mean | SD | P value |
| --- | --- | --- | --- |
| Sham | 4.0805 | 0.0596 | Sham VS OVX: 0.000 |
| OVX | 1.8888 | 0.6188 |  |
| OVX+L-THP | 3.4185 | 0.0856 | OVX VS OVX+L-THP: 0.01 |

**Figure 7A (BS/TV)**

| Group | Mean | SD | P value |
| --- | --- | --- | --- |
| Sham | 4.0275 | 0.1545 | Sham VS OVX: 0.001 |
| OVX | 1.6841 | 0.5299 |  |
| OVX+L-THP | 3.2172 | 0.3697 | OVX VS OVX+L-THP:0.01 |

**Figure 7A (BMD)**

| Group | Mean | SD | P value |
| --- | --- | --- | --- |
| Sham | 0.1047 | 0.0066 | Sham VS OVX: 0.000 |
| OVX | 0.6003 | 0.0070 |  |
| OVX+L-THP | 0.0965 | 0.0040 | OVX VS OVX+L-THP:0.001 |

**Figure 7B** (Tb.area)

| Group | Mean | SD | P value |
| --- | --- | --- | --- |
| Sham | 0.1368 | 0.0367 | Sham VS OVX: 0.01 |
| OVX | 0.0589 | 0.0155 |  |
| OVX+L-THP | 0.1206 | 0.0402 | OVX VS OVX+L-THP: 0.03 |

**Figure 7D** (TNF-α)

| Group | Mean | SD | P value |
| --- | --- | --- | --- |
| Sham | 19.8717 | 7.2086 | Sham VS OVX: 0.01 |
| OVX | 29.7175 | 5.9858 |  |
| OVX+L-THP | 20.7072 | 3.2712 | OVX VS OVX+L-THP: 0.01 |

**Figure 7D** (IL-6)

| Group | Mean | SD | P value |
| --- | --- | --- | --- |
| Sham | 7.8053 | 5.8159 | Sham VS OVX: 0.01 |
| OVX | 27.319 | 7.3672 |  |
| OVX+L-THP | 16.8153 | 7.0182 | OVX VS OVX+L-THP: 0.01 |

**Figure 7D** (CTX-1)

| Group | Mean | SD | P value |
| --- | --- | --- | --- |
| Sham | 44.2653 | 20.4112 | Sham VS OVX: 0.000 |
| OVX | 137.5558 | 37.2039 |  |
| OVX+L-THP | 56.9253 | 9.4212 | OVX VS OVX+L-THP: 0.000 |

**Figure 7D** (TRAcp5B)

| Group | Mean | SD | P value |
| --- | --- | --- | --- |
| Sham | 2.6003 | 0.4998 | Sham VS OVX: 0.000 |
| OVX | 4.6915 | 0.6478 |  |
| OVX+L-THP | 3.3685 | 0.2850 | OVX VS OVX+L-THP: 0.001 |

**Supplemental Figure S4** (RANK/β-actin)

| Group | Mean | SD | P value |
| --- | --- | --- | --- |
| Control | 1.00 | 0.06 | / |
| M-CSF | 1.63 | 0.10 | / |
| M-CSF+L-THP | 1.34 | 0.05 | / |
| M-CSF VS Control | / | / | <0.01 |
| M-CSF VS M-CSF+L-THP | / | / | <0.05 |

**Supplemental Figure S4** (c-fms/β-actin)

| Group | Mean | SD | P value |
| --- | --- | --- | --- |
| Control | 1.00 | 0.09 | / |
| M-CSF | 1.55 | 0.08 | / |
| M-CSF+L-THP | 1.30 | 0.15 | / |
| M-CSF VS Control | / | / | <0.01 |
| M-CSF VS M-CSF+L-THP | / | / | >0.05 |
